# Evaluating the use of serum total solids to predict serum immunoglobulin G in Holstein and Angus crossbred calves

**DOI:** 10.3168/jdsc.2025-0900

**Published:** 2026-02-13

**Authors:** Dini Hapukotuwa, John K. House, Katharine Denholm, Sam Rowe

**Affiliations:** 1 Sydney School of Veterinary Science, Faculty of Science, The University of Sydney, Camden, New South Wales 2570, Australia; 2 School of Biodiversity, One Health and Veterinary Medicine, University of Glasgow, Glasgow, Scotland G12 8QQ

## Abstract

This study evaluated the use of serum total solids (STS) to assess passive immunity in Holstein and Angus cross Holstein calves. The objectives of our study were to (1) determine whether STS and serum IgG differs between Holstein and Angus × Holstein (Angus X) newborn calves, (2) compare the relationship between STS and serum IgG in both breeds, and (3) determine whether the industry-recommended passive immunity STS cutpoint (>62 g/L) for predicting serum IgG concentration ≥25 g/L is appropriate for Angus X calves. This observational study was part of a wider clinical trial in which calves were randomized to colostrum IgG doses ranging from 200 to 350 g. Blood samples were taken from calves at 2 d of age, and the separated serum was tested for STS (g/L) using optical refractometry. The remaining serum was stored frozen at −20°C and tested 2 wk later for IgG concentration (g/L) using radial immunodiffusion. Multivariable linear regression models were used to compare serum IgG and STS between breeds, controlling for colostrum IgG dose and calf sex. Pearson correlation coefficient and linear regression models were used to compare correlations between serum IgG and STS by breed. Area under the curve (AUC) and diagnostic sensitivity (Se) and specificity (Sp) were used to measure the ability of STS to identify calves with serum IgG ≥25 g/L. The number of calves analyzed in the final dataset from each group were 272 Holstein and 119 Angus X. Although serum IgG was similar between breeds, Holstein calves had 3.1 g/L (95% CI: 1.7, 4.5) higher STS than Angus X calves. The AUC of STS for predicting IgG ≥25 g/L was the same for both breed groups (AUC = 0.86). A previously recommended cutpoint of 62 g/L STS had Se and Sp of 51% and 91%, respectively, when predicting serum IgG ≥25 g/L in Holsteins. We found that a STS cutpoint that was 3 g lower (59 g/L) in Angus X calves provided similar performance (Se 55%, Sp 94%). Although colostral IgG absorption is similar between breeds, the relationship between serum IgG and STS differs, likely due to Angus X calves having a lower concentration of non-IgG proteins in serum. Although we acknowledge that the sensitivity of these cutpoints is low, we believe that breed-specific thresholds may be necessary to predict a calf serum IgG ≥25 g/L for Angus X calves, compared with the >62 g/L recommendation proposed for Holsteins in the literature.

## Highlights

• The correlation between serum IgG and STS was similar between breeds.
• Diagnostic accuracy for predicting serum IgG ≥ 25 g/L using STS was similar between breeds.
• Holstein calves had higher STS compared with Angus X calves, despite similar serum IgG.
• When using STS to estimate serum IgG in calves, breed-specific cutpoints should be used.

## Graphical Abstract

**Figure:**
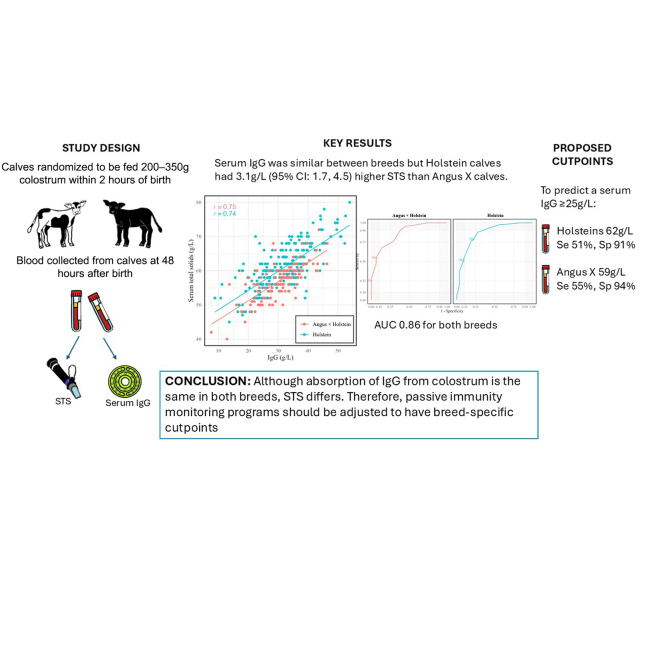
**Summary:** Serum total solids (STS) measured by optical or digital refractometry is a rapid and inexpensive method to indirectly measure immunoglobulin G (IgG) concentration in serum, and is commonly used to monitor the transfer of passive immunity on farms. However, most cutpoints used in practice have been developed from Holstein calf data, and their application to dairy × beef crossbred animals is uncertain. This observational study compared the STS and serum IgG concentration in 272 Holstein and 119 Angus × Holstein (Angus X) calves at 48 hours of age. Serum IgG was similar between breeds, but Holstein calves had, on average, higher STS values compared with Angus X calves. Therefore, it was determined that breed-specific cutpoints are required to accurately identify calves with excellent passive immunity (serum IgG ≥25 g/L). The optimal cutpoints determined for each breed were 62 g/L for Holsteins and 59 g/L for Angus X calves. AUC = area under the curve.

Calves are an essential part of dairy farming because they are the future of the milking herd and the key to genetic improvement (Palczynski et al., 2022). However, calf disease can have a significant impact on welfare, survival, long-term productivity (Goh et al., 2024), and farm finances, costing an estimated US$148 (Rocha Valdez et al., 2019) per case of diarrhea alone. Because calves are born agammaglobulinemic (Morin et al., 1997), colostrum IgG ingested at birth is critical for providing adequate immunity. The IgG absorbed into the calf's serum is protective against disease and death. United States colostrum experts have recently created new target categories of passive immunity (measured by serum IgG): “poor,” <10 g/L; “fair,” 10 to 17.9 g/L; “good,” 18 to 24.9 g/L; and “excellent,” ≥25 g/L (Lombard et al., 2020), where calves falling into each increasing passive immunity category have decreased risk of morbidity and mortality. According to Lombard et al. (2020), producers should aim to have >40% of calves in the excellent passive immunity category and <10% of calves in the poor category, also referred to as failure of transfer of passive immunity (**FTPI**). However, direct measurement of IgG by farmers or their advisors is rarely practical, given the cost, complexity, and time-consuming nature of using laboratory assays such as radial immunodiffusion (**RID**).

Optical or digital refractometry is a rapid and inexpensive method to measure the total solids in a solution. In a neonatal calf, the predominant solid present in the calf's serum is protein, primarily IgG (Thompson and Smith, 2023). Therefore, STS by refractometry is often used indirectly to measure IgG concentration in serum and is commonly used to monitor transfer of passive immunity (**TPI**) on farms (Deelen et al., 2014). Multiple studies have evaluated the accuracy of STS as a predictor of calf serum IgG (Deelen et al., 2014; Hernandez et al., 2016; Elsohaby et al., 2019) and showed good correlation, with Pearson correlation coefficient values ranging between 0.79 and 0.93. These studies compare the ability of STS to predict FTPI in calves. The sensitivities (**Se**), and specificities (**Sp**), and area under the curve (**AUC**) for these studies were Se 76.3, Sp 94.4 at 55 g/L STS (Deelen et al., 2014); Se 100, Sp 80.4, and AUC 0.95 at STS 52 g/L (Hernandez et al., 2016); and Se 75.5, Sp 77.2, and AUC 0.87 at 51 g/L STS (Elsohaby et al., 2019). However, there is no current published literature that measures the Se and Sp of proposed STS cutpoints used to predict serum IgG concentrations of ≥25 g/L in Holstein calves (Lombard et al., 2020). This highlights a gap in the literature that requires further exploration should this cutpoint be used to aim for excellent TPI. However, in beef cattle, Akköse et al. (2023) explored the diagnostic performance of STS to predict a serum IgG of <24 g/L in Aberdeen Angus and Limousin calves and found that a <64 g/L STS cutpoint had a Se of 87.5, Sp of 69.7, and AUC of 0.89. Nevertheless, the predictive ability of STS is generally not sufficient to make individual animal decisions given that Se and Sp are imperfect but can be used to detect population-level trends over time for management decisions.

Limitations in the use of STS as a proxy for IgG concentration may be due the presence of other immunoglobulins (e.g., IgM and IgA) or non-IgG proteins present in serum, such as albumins, haptoglobin, transferrin, and lactoferrin (Tóthová et al., 2016b), which may vary within a population of calves, although are generally considered more stable compared with IgG concentrations (Tóthová et al., 2016a). Additionally, evidence exists to suggest that STS are lower in calves fed colostrum replacers, despite achieving an adequate level of passive immunity (Quigley et al., 2002). However, it must be understood that STS cutpoints to predict serum IgG values in calves fed colostrum replacers will vary among different products as a result of different product manufacturing methods and resultant non-IgG solids composition. Breed differences in STS have also been described. For example, Immler et al. (2022) found differences in STS for Holstein calves when compared with the Brown Swiss and Simmental calves. In that study, no significant differences in serum IgG were found, suggesting that the difference in STS was due to differing concentrations of non-IgG proteins. This difference may be due to different serum albumin concentrations, as studies in adult cattle report breed variations whereby Jersey cattle have been found to have higher average serum albumin concentrations in comparison to Guernsey, Holstein Friesian, and Brown Swiss breeds (Shaffer et al., 1981). Bobbo et al. (2017) also reported that Jersey cattle had the highest serum albumin concentrations when compared with Brown Swiss, Holstein Friesian, Simmental, Rendena, and Alpine Grey breeds.

Most studies use data from Holstein calves when developing STS cut-off values for monitoring passive immunity (Deelen et al., 2014; Elsohaby et al., 2019). And although our previous investigation demonstrated that colostrum IgG dosing for Angus X and Holstein calves should be similar (Hapukotuwa et al., 2026), it is possible that using the same STS cutpoints for monitoring of passive immunity in both breeds is inappropriate.

With dairy × beef becoming a growing market in the dairy industry, a need exists for further investigation into the use of STS as a tool for predicting serum IgG concentration in dairy × beef calves. The objectives of our study were to (1) determine whether STS and serum IgG differ between Holstein and Angus X newborn calves (independent of colostrum feeding), (2) compare the relationship between STS and serum IgG in both breeds, and (3) determine whether the industry-recommended passive immunity STS cutpoint (>62 g/L) for predicting serum IgG concentration ≥25 g/L is appropriate for Angus X calves.

This observational study was conducted within a clinical trial involving 400 (274 Holstein and 126 Angus X) calves on a dairy farm in southern Australia (Hapukotuwa et al., 2026). The sample size for Holstein calves was calculated for the clinical trial based on an expected difference in serum IgG of 4 g/L among oral doses of 200 and 250 g of IgG, assuming an SD of 5, delta of 0, power of 0.8, and an α level of 0.05 requiring a minimum sample size of 70 calves per treatment group. Therefore, data regarding 400 calves (including Angus X calves and the unplanned 350 g colostrum dose group) were available for inclusion in the present study. Ethics approval for the clinical trial was granted by the University of Sydney animal ethics committee (2024/AE002408).

Colostrum was harvested from cows within 2 h of birth using a portable milking machine (iMilk 2.0 [Double], Daviesway DASCO Pty Ltd.), pooled, heat-treated in a pasteurizer system (Perfect Udder Combi Pasteurizer, Dairy Tech Inc.) for 60 min at 60°C and then cooled to 32°C and stored refrigerated at 2°C. A 30-mL aliquot was taken to test the colostrum concentration via RID (Bovine IgG Test Kit, Triple J Farms, Bellingham, WA) on farm. Once the concentration of colostrum IgG was determined 24 h later, the required volume for each dose was calculated by referring to a dosage table that showed volumes of each dose for colostrum concentrations ranging from 50 to 200 g/L. The stored colostrum of the tested batch was mixed thoroughly and then measured out into the required volumes using a 5-L graduated jug to create doses of 200, 250, 300, or 350 g of IgG.

Calves were ID tagged (based on breed) in sequential order and were randomized by ID tag number before birth to receive colostrum doses ranging from 200 to 350 g of IgG. There were 2 randomization periods in the study. The first period involved block randomization (block size 6, ratio of 1:1:1, sequence determined using a random-number generator) of 200-, 250- and 300-g doses to calves across 7 d. However, farm managers on the enrolled farm became concerned when routine monitoring found that average STS had dropped during the first week of the trial. Consequently, no additional calves were enrolled into the lowest dose group (200 g), and a second randomization period commenced, with doses adjusted so that the remaining calves received doses of 250, 300, and 350 g at an enrollment ratio of 2:2:3, respectively using a block size of 7 across 11 d. Because the overall proportion of Angus X calves born during the first week of the enrollment period was greater (56 born out of 126 total) than that of Holstein calves (74 born out of 274 total), the subsequent proportion of Angus X calves randomized to the 200-g group during the first week was higher (17.6%) than that of Holstein calves (8.8%). Colostrum IgG dose group (g) was later controlled for in our analysis (see statistical methods). Any calf born without assistance was enrolled into the study. Animals were excluded if farm staff failed to record the unique colostrum dose ID that was administered (n = 6). Calves were fed within 2 h of birth via esophageal tube and blood was collected from calves by jugular venipuncture at 2 d of age using an 18-gauge needle and plain serum Vacutainer blood collection tubes (BD Vacutainer, Sydney, NSW, Australia).

The main variables of interest were STS (g/L) and serum IgG concentration (g/L). The STS were measured by centrifugation (3,000 × *g* for 5 min at 24°C) of the 2-d blood samples and placing 0.3 mL onto a calibrated optical refractometer (Trusti; Shoof Direct, Tullamarine, Victoria). This procedure was part of the normal farm passive immunity monitoring program. Once on-farm testing was completed, the remaining serum was collected using a Pasteur pipette and placed into a sterile 5-mL sample vial, labeled with calf ID, and stored at −20°C for IgG testing at the University of Sydney internal laboratory.

Serum IgG concentration was tested using RID. The RID plates were acclimatized to room temperature for 30 min and serum samples were thawed to 37°C in a water bath. The reference standards of 280, 700, 1,400, 1,800, and 2,800 mg/dL were tested in triplicate, and the results were used to generate a standard curve for each testing batch. Serum samples were vortexed, diluted 1:1, and tested in duplicate by loading 5 µL per sample well. The RID plates were incubated at 20°C to 24°C for 24 h in a plastic container lined with damp paper towels to create a moist chamber. Digital calipers were used to measure precipitation ring diameters in duplicate to the nearest 0.1 mm. Ring measurements were averaged, squared, and converted to IgG concentrations using the standard curve generated from the reference sera. Reference standards were performed for each RID testing batch for calf serum.

All statistical analyses were conducted using R statistical software (R Core Team, 2018). An analysis log can be found in Supplemental File S1 (see Notes). Analysis aimed to answer the following questions:

1. Does breed affect serum IgG or STS, or both?
2. Does the correlation between serum IgG and STS differ between breeds?
3. What STS cutpoints provide the best prediction of excellent TPI (IgG concentration ≥25 g/L) in Angus X calves?

Multivariable linear regression was performed to estimate the effect of breed on serum IgG, and on STS. Both models included IgG dose group and calf sex as covariates. A causal diagram (directed acyclic graph, shown in the analysis log, see Notes) was used to decide which covariates were included in the final model (VanderWeele et al., 2021). Because the Angus X calves were born at a greater rate during the first randomization period, they were more likely than Holstein calves to receive the 200-g dose (relative risk = 2.0) and less likely to receive the 350-g dose (relative risk = 0.7). Consequently, the model controlled for IgG dose because it was associated with the exposure of interest (breed) and the outcome (serum IgG concentration). We also adjusted for calf sex to estimate the direct effect of breed that was not explained by the higher prevalence of heifer calves in the Holstein group. Sensitivity analyses were conducted to identify whether conclusions would be different if other variables were included in the model. Model-adjusted mean IgG and STS were calculated for each breed (estimated marginal means). To assess the correlation between STS and IgG, we generated breed-stratified regression lines and calculated Pearson correlation coefficient values. To assess the diagnostic capability to predict calves with a serum IgG ≥25 g/L, receiver operating characteristic (**ROC**) curves were drawn, and test performance measures were calculated, including AUC, diagnostic Se, and Sp.

A total of 391 serum samples were tested from Holstein (n = 272) and Angus X (n = 119) calves after accounting for animals lost to follow up (n = 1 Holstein) and testing error (n = 4 Holstein, n = 4 Angus X). Because 2 randomization periods involving different treatment doses were included, and there were more Angus X calves being born and subsequently enrolled during the first randomization period, the Angus X calves had overall lower colostral IgG doses on average (SD) compared with Holstein calves: 278 g (50.3) versus 290 g (48.4). This was because of more 200-g doses being delivered to Angus X calves during the initial randomization period. The proportion of calves falling into the proposed serum IgG categories as reported by Lombard et al. (2020) for Holstein and Angus X calves, respectively, were 80.6% and 73.7% for ≥25 g/L, 16.1% and 21.2% for 18 to 24.9 g/L, 2.9% and 4.2% for 10 to 17.9 g/L, and 0.4% and 0.8% for <10 g/L. The median [min, max] for other variables associated with STS in our causal diagram include time to feeding (50.0 [0, 190] min for Angus X and 50.0 [0, 195] min for Holstein calves), volume at feeding (3.10 L [1.80, 3.90] for Angus X and 3.10 L [1.80, 3.90] for Holstein calves), and calf sex (n (%); 60 (50.4%) female and 59 (49.6%) male Angus X and 255 (93.8%) female and 17 (6.3%) male Holstein calves). The higher proportion of male calves in the Angus X group was because of the use of sexed female Holstein and nonsexed Angus semen straws in the farm's breeding program.

The mean (SD) serum IgG was 32.1 g/L (8.0) and 30.2 g/L (7.3) for Holstein and Angus X calves, respectively. The mean (SD) STS was 61.0 g/L (6.1) and 56.9 g/L (5.47) for Holstein and Angus X calves, respectively (Figure 1). The multivariable linear regression for the effect of breed on serum IgG found that Holsteins (30.3 g/L) and Angus X (30.0 g/L) had similar estimated marginal mean IgG (+0.3 g/L 95% CI [−1.5, 2.1] for Holsteins) after adjusting for colostral IgG dose and calf sex. The same analysis performed to measure the effect of breed on STS showed a difference between breeds, with Holstein model-adjusted mean STS (60.0 g/L) being 3.1 g/L 95% CI [1.7, 4.5] higher than Angus X (56.9 g/L, Figure 1). These findings were consistent when time to feeding and volume were included in sensitivity testing models. Other studies have also found breed differences in STS (Villarroel et al., 2013; Immler et al., 2022). Immler et al. (2022) found that STS in Holstein calves was different that in Brown Swiss or Simmental calves, but that serum IgG was more similar among breeds. Villarroel et al. (2013) found that Jersey calves had consistently higher STS than Holsteins, being on average 4.9 g/L higher during the first week of life. They also found that Jersey calf serum IgG was on average 19.9 g/L higher than that of Holstein calves during the first week of life. However, it should be noted that in these studies, colostrum IgG dose was not controlled for, leading to differences in STS being potentially attributable to colostrum feeding practices rather than true breed differences.

**Figure 1:**
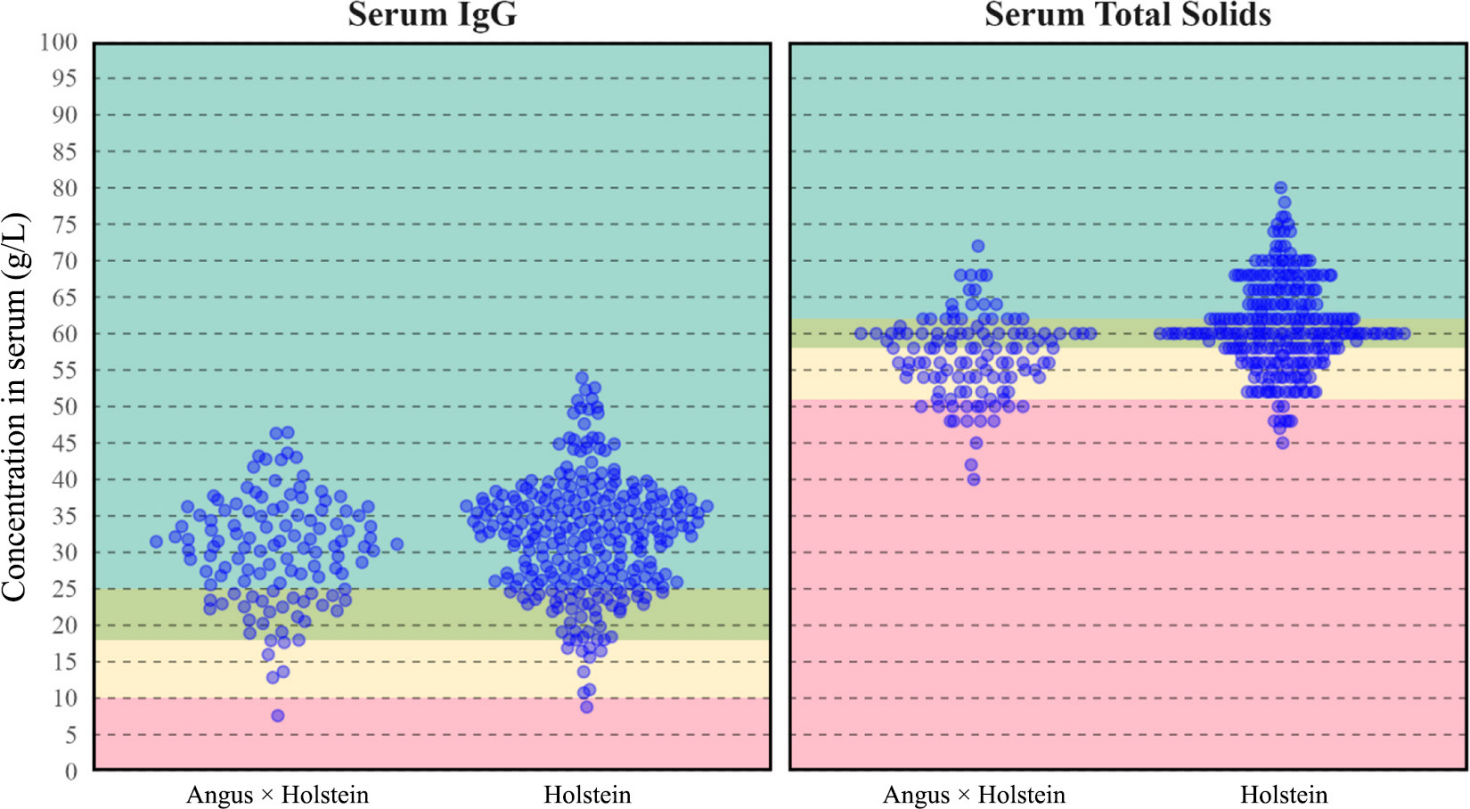
Scatterplot of calf serum values of IgG and total solids stratified by breed, Holstein × Angus (n = 119) and Holstein (n = 272), in an observational study comparing serum total solids (STS) and serum IgG in Australian calves. Each dot represents a calf serum value of either IgG (left) or total solids (right). The background colors represent proposed serum IgG (left) and STS (right) cutpoints for passive immunity based on current recommendations by US colostrum experts (Lombard et al., 2020). These are excellent ≥25 g/L (teal); good, 18 to <25 g/L (olive); fair 10 to <18 g/L (yellow); and poor <10 g/L (pink) for serum IgG and excellent, ≥62 g/L (teal); good, 58 to 61 g/L (olive); fair, 51 to <57 g/L (yellow); and poor, <51 g/L (pink) for STS.

We hypothesize that the breed difference in STS in our study was due to lower concentrations of non-IgG proteins in Angus X serum, particularly as our comparison was adjusted for oral IgG dose. A likely candidate protein is albumin. This hypothesis is supported by observational studies in adult cattle (Shaffer et al., 1981; Bobbo et al., 2017). Bobbo et al. (2017) found that Jersey cows had higher serum albumin concentrations when compared with that of Brown Swiss, Holstein Friesian, Simmental, Rendena, and Alpine Grey breeds managed across 41 multibreed farms. Shaffer et al. (1981) also found that Jersey cattle had the highest mean serum albumin (33.5 g/L) compared with Guernsey, Holstein Friesian, and Brown Swiss breeds (31.2, 29.6, and 32.2 g/L). More research is needed to investigate this hypothesis.

The correlation between serum IgG and STS was similar between breeds (Figure 2). This was evidenced by the finding of similar Pearson correlation coefficient values for Holstein and Angus X (r = 0.74 versus 0.75, respectively) and regression slopes (0.56). Receiver operating characteristic curves were constructed for each breed STS value to determine the optimal cutpoint for predicting a serum IgG at ≥25 g/L, classed as an excellent level of passive immunity by the newest recommendations in dairy calves (Lombard et al., 2020). The AUC for both breeds was 0.86. The 62 g/L STS cutpoint for Holstein calves had an Se of 51% and Sp of 91%. In this dataset the diagnostic test performance in Angus X calves showed minor improvement when lowering the test cutpoint from 62 g/L (Se = 25%, Sp = 100%) to 59 g/L (Se = 55%, Sp = 94%; Figure 3), the lower cutpoint showing diagnostic performance similar to the 62-g/L recommendation that exists in Holstein calves. Therefore, for herds with Angus X calves that want to monitor serum IgG ≥25g/L by STS, a cutpoint of 59 g/L may achieve similar diagnostic performance to what has been recommended previously in Holstein calves. We did not evaluate the use of STS to predict IgG concentrations <10 g/L because very few of the calves (n = 2) fell into this category.

**Figure 2:**
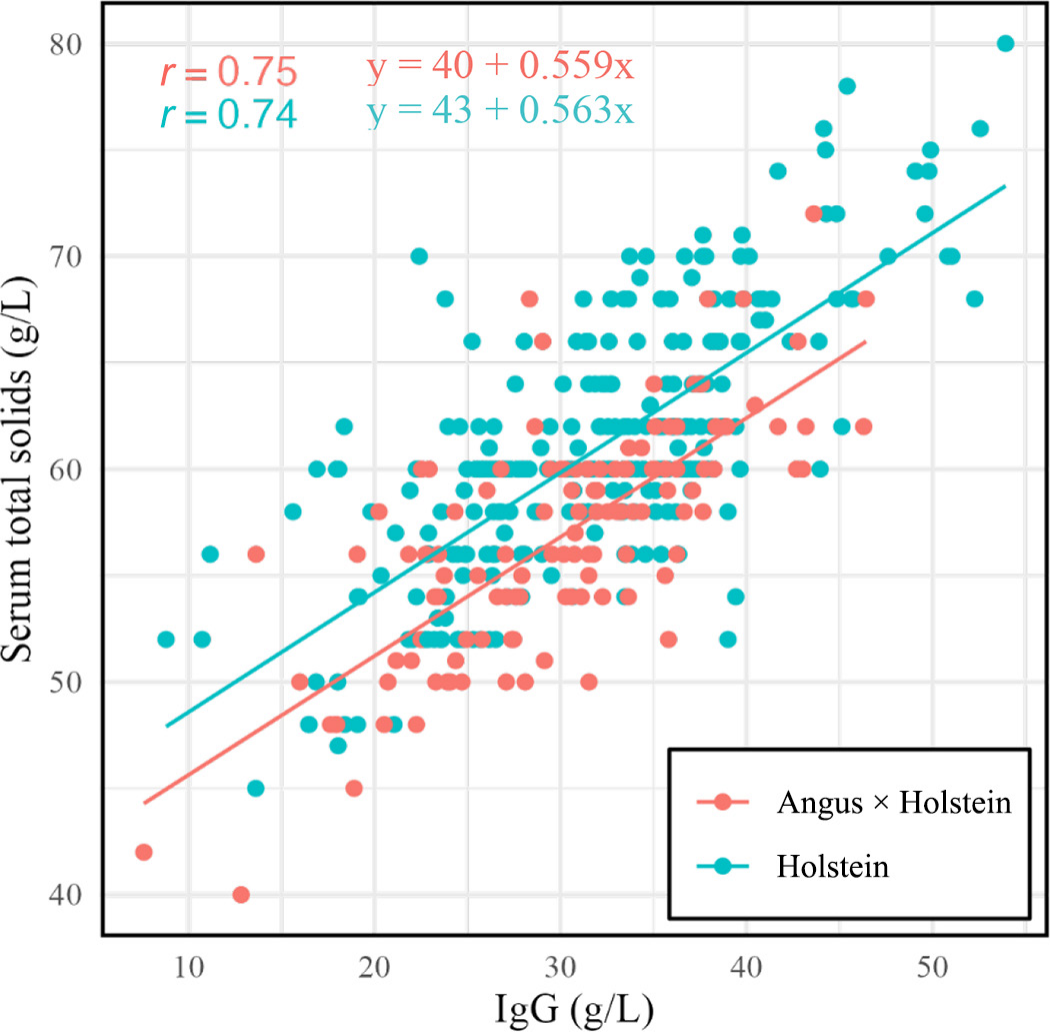
Scatterplot showing the correlation between STS and serum IgG, stratified by Holstein (teal) and Angus X Holstein (red) breeds in an observational study comparing STS and serum IgG in Australian calves. The linear regression line parameters are shown. The slope of both lines was the same (0.56), despite being determined independently, indicating that for every 1 g/L increment in STS, IgG concentration increased by 0.56 g/L. Pearson correlation coefficient values (r) were numerically similar for Holstein (0.74) and Angus X calves (0.75).

**Figure 3:**
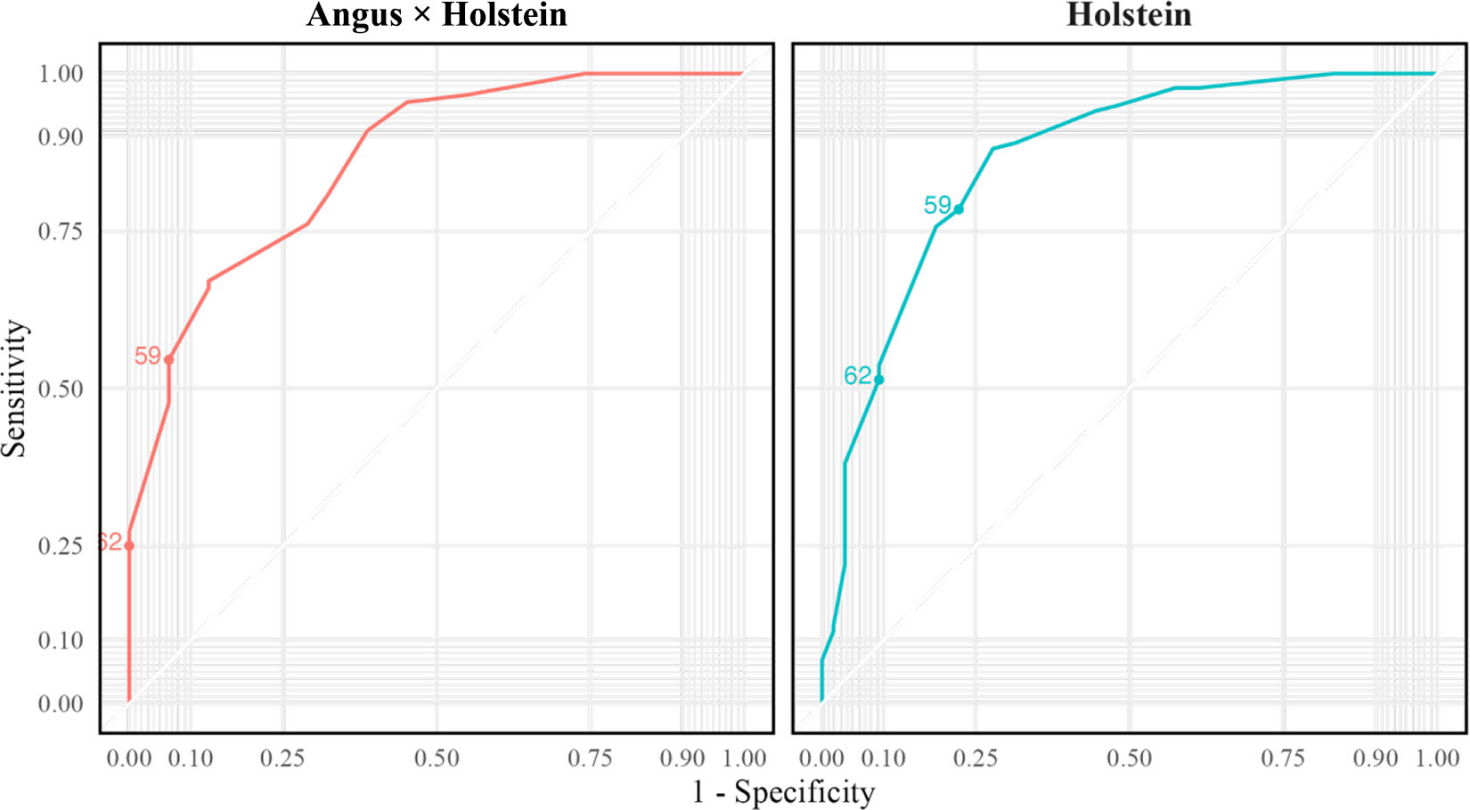
Receiver operating characteristic curve assessing the use of STS to predict a serum IgG ≥25 g/L at various cutpoints for each breed (Holstein, right and Angus X Holstein, left). The AUC for both curves was 0.86. Data were derived from an observational study evaluating the relationship between STS and serum IgG concentrations in Australian dairy and dairy × beef crossbred calves.

To our knowledge, there are no previous studies comparing the Pearson correlation of IgG and STS between different breeds. Most studies report correlations solely for Holstein calves, with Pearson correlation values ranging between 0.75 and 0.93 (Deelen et al., 2014; Hernandez et al., 2016; Elsohaby et al., 2019). In studies assessing passive immunity in beef suckler calves, Pearson correlation coefficients for the association between serum IgG and STS have been reported to be from 0.64 (Todd et al., 2018) to 0.87 (Akköse et al., 2023). The latter study also found a similar AUC for predicting a serum IgG <24 g/L in beef calves (0.89), as in the present study. Our finding that STS measurement as a proxy for serum IgG should be considered on a breed-specific level is supported by Villarroel et al. (2013), who found that different cutpoints to determine a serum IgG concentration of <10 g/L were required for Jersey (43 g/L) and Holstein (51 g/L) calves.

A major study strength is that both breeds in this study were exposed to the same colostrum-management protocol, which was randomized to calves. Potential confounding was minimized in the statistical analysis by using multivariable models that adjusted for differences in colostrum IgG dose to allow for an unbiased comparison between breeds.

The main limitation of our study was that this was a single-herd study, and despite the objectives primarily focusing on the relationship between serum IgG, STS, and breed alone, we recognize that data from a single farm will reduce the external validity of our findings to other farms. It is also noted that we were only able to evaluate Holstein and Angus × Holstein calves present on this farm, which limits the application of our findings to other breeds. Furthermore, there were very few calves in our study that had FTPI (serum IgG <10 g/L, n = 2), and therefore we were unable to evaluate the performance (i.e., Se and Sp) of STS to identify calves with FTPI. We also acknowledge that although STS was conducted on fresh serum samples immediately after collection, RID was conducted using frozen samples that were tested between 9 and 54 d after collection.

Future studies should focus on evaluation of other dairy breeds as well as crossbred calves because dairy × beef crossbred calves are becoming increasingly common in the dairy industry. Additionally, a multiherd study, including herds with a higher prevalence of FTPI will aid in the prediction of low serum IgG.

## References

[bib1] Akköse M., Buczinski S., Özbeyaz C., Kurban M., Cengiz M., Polat Y., Aslan O. (2023). Diagnostic accuracy of refractometry methods for estimating passive immunity status in neonatal beef calves. Vet. Clin. Pathol..

[bib2] Bobbo T., Fiore E., Gianesella M., Morgante M., Gallo L., Ruegg P., Bittante G., Cecchinato A. (2017). Variation in blood serum proteins and association with somatic cell count in dairy cattle from multi-breed herds. Animal.

[bib3] Deelen S.M., Ollivett T.S., Haines D.M., Leslie K.E. (2014). Evaluation of a Brix refractometer to estimate serum immunoglobulin G concentration in neonatal dairy calves. J. Dairy Sci..

[bib4] Elsohaby I., McClure J., Waite L., Cameron M., Heider L., Keefe G. (2019). Using serum and plasma samples to assess failure of transfer of passive immunity in dairy calves. J. Dairy Sci..

[bib5] Goh N., House J., Rowe S. (2024). Retrospective cohort study investigating the relationship between diarrhea during the preweaning period and subsequent survival, health and production in dairy cows. J. Dairy Sci..

[bib6] Hapukotuwa D.A., House J.K., Denholm K., Rowe S. (2026). Randomized clinical trial evaluating the practical and biological limits of colostral immunoglobulin G dosing in Holstein and Angus X calves. J. Dairy Sci..

[bib7] Hernandez D., Nydam D., Godden S., Bristol L., Kryzer A., Ranum J., Schaefer D. (2016). Brix refractometry in serum as a measure of failure of passive transfer compared to measured immunoglobulin G and total protein by refractometry in serum from dairy calves. Vet. J..

[bib8] Immler M., Büttner K., Gärtner T., Wehrend A., Donat K. (2022). Maternal impact on serum immunoglobulin and total protein concentration in dairy calves. Animals (Basel).

[bib9] Lombard J., Urie F., Garry S., Godden J., Quigley T., Earleywine S., McGuirk D., Moore M., Branan M., Chamorro G., Smith C., Shivley D., Catherman D., Haines A.J., Heinrichs R., James J., Maas J., Sterner K. (2020). Consensus recommendations on calf- and herd-level passive immunity in dairy calves in the United States. J. Dairy Sci..

[bib10] Morin D.E., McCoy G.C., Hurley W.L. (1997). Effects of quality, quantity, and timing of colostrum feeding and addition of a dried colostrum supplement on immunoglobulin G_1_ absorption in Holstein bull calves. J. Dairy Sci..

[bib11] Palczynski L.J., Bleach E.C., Brennan M.L., Robinson P.A. (2022). Youngstock management as “The key for everything”? Perceived value of calves and the role of calf performance monitoring and advice on dairy farms. Front. Anim. Sci..

[bib12] Quigley J.D., Kost C.J., Wolfe T.M. (2002). Absorption of protein and IgG in calves fed a colostrum supplement or replacer. J. Dairy Sci..

[bib13] R Core Team (2018). https://www.r-project.org/.

[bib14] Rocha Valdez J., Gonzalez-Avalos R., Avila-Cisneros R., Peña-Revuelta B., Reyes-Romero A. (2019). Economic impact of mortality and morbidity from diseases in dairy calves. Abanico veterinario.

[bib15] Shaffer L., Roussel J., Koonce K. (1981). Effects of age, temperature-season, and breed on blood characteristics of dairy cattle. J. Dairy Sci..

[bib16] Thompson A.C., Smith D.R. (2023). Estimating IgG concentration directly by radial immunodiffusion or indirectly by refractometry measure of serum total protein lack precision. Am. J. Vet. Res..

[bib17] Todd C.G., McGee M., Tiernan K., Crosson P., O'Riordan E., McClure J., Lorenz I., Earley B. (2018). An observational study on passive immunity in Irish suckler beef and dairy calves: Tests for failure of passive transfer of immunity and associations with health and performance. Prev. Vet. Med..

[bib18] Tóthová C., Nagy O., Kováč G., Nagyová V. (2016). Changes in the concentrations of serum proteins in calves during the first month of life. J. Appl. Anim. Res..

[bib19] Tóthová C., Nagy O., Nagyová V., Kováč G. (2016). The concentrations of selected blood serum proteins in calves during the first three months of life. Acta Vet. Brno.

[bib20] VanderWeele T.J., Rothman K.J., Lash T.L. (2021). Modern Epidemiology.

[bib21] Villarroel A., Miller T., Johnson E., Noyes K., Ward J. (2013). Factors affecting serum total protein and immunoglobulin G concentration in replacement dairy calves. J. Adv. Dairy Res..

